# Analysis of the Influence of the GPS Errors Occurred While Collecting Electrode Coordinates on the Electrical Resistivity of Tumuli †

**DOI:** 10.3390/s20102966

**Published:** 2020-05-23

**Authors:** Veronica Pazzi, Mattia Ceccatelli, Lorenzo Ciani, Gabriele Patrizi, Giulia Guidi, Luca Cappuccini, Nicola Casagli, Marcantonio Catelani

**Affiliations:** 1 Department of Earth Sciences, University of Florence, 50121 Florence, Italy; mattia.ceccatelli@unifi.it (M.C.); Nicola.casagli@unifi.it (N.C.); 2 Department of Information Engineering, University of Florence, 50139 Florence, Italy; lorenzo.ciani@unifi.it (L.C.); gabriele.patrizi@unifi.it (G.P.); giulia.guidi@unifi.it (G.G.); marcantonio.catelani@unifi.it (M.C.); 3 Department of History, Archaeology, Geography, Fine and Performing Arts, University of Florence, 50121 Florence, Italy; luca.cappuccini@unifi.it

**Keywords:** funeral mound, Monte Carlo simulation, geophysical method, electrode-spacing error, electrical resistivity tomography, archaeo-geophysics, archaeology

## Abstract

In archaeological applications the accurate reconstruction of buried structures is mandatory. Electrical resistivity tomography is widely used for this purpose. Nevertheless, resistivity errors could be generated by wrong placement of electrodes. Papers in the literature do not discuss the influence of errors connected with the electrode position location (GPS-error). In this paper the first results of a Monte Carlo simulation analysis of data acquired on a tumulus are presented. The main research questions were: (i) if it is correct to ignore the GPS-error collect, and (ii) if a minimum threshold, that significantly affect the inversion, exists. Results, obtained considering planimetric GPS-errors of about one third of the fixed electrode distances, show that the GPS-errors affect resistivity, but the generated errors/anomalies: (a) are lower than that obtained without considering the topography, and (b) are significant from a numerical point of view, but do not affect the interpretation, being compatible with the soil resistivity ranges.

## 1. Introduction

Electrical resistivity tomography (ERT) is a geophysical technique widely used in environmental investigations [1,2,3,4,5,6,7,8], hydrogeology [9,10,11], civil engineering [12,13], investigations on waste disposal sites [14,15], and archaeology [16,17,18,19]. 2D and 3D geophysical investigation of tumuli is a very challenging field of application, as a consequence of the prevailing rough topography conditions and complex subsoil resistivity distribution. Ancient buried anthropogenic structures are usually resistive, have dimensions of a few meters, and are generally located at low depths from the ground surface [18,19,20,21,22]. Since its first development, despite the successes of the ERT method, quality data (noise contamination/high signal to noise ratio) has affected the technique results resolution and reliability [14,23,24,25]. Moreover, in archaeological applications, the accurate resolution in identifying the dimensions and the depth of buried objects strongly depends on the electrodes’ spatial distribution [3,17,18]. Therefore, it is important to avoid as much as possible errors induced by inaccurate electrode positioning.

Zhou and Dahlin [25] reported that errors that affect ERTs could be classified in two categories: measured voltage errors and electrode-spacing errors (called “mislocation” in [23]). Voltage errors are caused by many factors, i.e., ineffective electrode contacts, damages to the cables or problems in the resistivity meter and background noises. In particular, potential errors are inversely proportional to the potential measured at the voltage electrodes. Therefore, this kind of error is mainly related to the strength of the input signal [25].

The second kind of error is caused by a wrong positioning of the electrodes and/or by ignoring the topography in the inversion procedure [23,24,25,26,27,28,29,30]. In particular, a wrong electrode positioning can occur: (a) when electrodes are set up by means of a tape or a string (and therefore a human error can occur), (b) when the interelectrode distance is equal to the cable fixed-space between two connections, but the cable could not be properly stretched because of the topography conditions and the terrain roughness, (c) when some electrodes have to be moved from their theoretical position to be better coupled with the soil (because of the topography and/or the presence of rocks), and d) when the finite cable length does not allow one to place the remote pole/s at the theoretical infinite distance (according to the chosen array). This kind of error could be reduced by paying attention during the field work but cannot be totally avoided. Moreover, in borehole surveys, errors related to a mislocation of the electrodes could occur if the boreholes themselves are deviated [15,23]. Finally, in Uhlemann et al. [8] a case study of time-lapse geoelectrical monitoring of an active landslide is presented. They showed the importance of knowing the exact position of the electrodes to avoid misinterpretations of the resistivity anomalies that are caused by the electrode’s movements along the landslide.

Zhou and Dahlin [25] stated that an electrode positioning error of 10% could happen when the electrode distance is less than 2 m. Oldenborger et al. [23] quantified, by means of a sensitivity function, the dependence of data on the electrode-spacing error. They also calculated a total systematic error considering one electrode affected by a position error and all the potential field perturbations coupled with all the quadripoles associated with the electrode. Even a generalization of the distribution is difficult, in the last two mentioned papers is shown how the errors in locating electrodes can generate artefacts in the acquired data and in the inverted model. Before these two works, Morelli and LaBrecque [31], studying the ERT robust inversion of crosshole data, already demonstrating how a wrong electrode position can affect the acquired and/or inverted resistivity data generating artefacts. At the contrary, Szalai et al. [24] proven how induced artifacts caused by an electrode-spacing error are not so significant, apart in rocky terrain, where the error can be maintained low, preferring off-line (i.e., perpendicular to the survey line) electrode displacements than in-line ones (i.e., along the survey line). More recently, Clement and Moreau [14] conducted a study at laboratory scale demonstrating that the influence on the geometric factor of the electrode diameter and embedment is negligible, while that of the electrode-spacing error is considerable. Moreover, they showed that vertical arrays are more affected by errors in electrodes positions than horizontal arrays. Since their results are considered to be also valid at field scale, when the inter-electrode space is lower than 1 m, it could be interpreted that boreholes measurements are more sensitive to the electrode-spacing errors than surface ones, in agreement with literature discussed above.

In the earth sciences field the electrode-spacing error is not always taken into account during field work, and therefore, it is a quite often an overlooked problem. Nevertheless, each kind of array is affected in a different way by the errors occurred in a wrongly placement of at least one electrode [14,23,24,25,29]. First of all, the electrode-spacing error is higher in arrays with a smaller distance among the electrodes because the percentage error is higher [14,15,24]. Then, the dipole-dipole (DD) array is the most sensitive to mispositioning errors [14,25] because the changes of its geometric factor are proportional to the cube of the distance between current and potential dipoles. In a pole-dipole (PD) array, electrode-spacing errors can also be caused by the position of the remote pole, that is commonly assumed to be located at an infinite distance from the ERT line [26,27,29,30]. Razafindratsima and Lataste [29] discussed the results of inverted models and field surveys performed to evaluate the errors in apparent resistivity and geometric factor using both the infinite theoretical distance and the finite real position of a remote electrode. They concluded that it is always better to use the real remote electrode position instead of putting it at theoretic infinite distance.

The anomalies caused by electrodes’ misplacements, as well as errors due to the topography effect, can be partially avoided thanks to the improvement of the technology. Nowadays, in fact, it is possible to measure the real/actual electrode position with a very high precision, i.e., by means of a laser distance meter [24] or by means of Differential Global Position System (DGPS) instruments [4,8,32,33,34]. Modern inversion software packages are able to take into account the real positions of electrodes [13,28,30] and, therefore, to calculate the correct geometric factor of an ERT acquisition. It is important to point out that a GPS system acquires the electrode’s position coordinates with a certain precision/error degree (in the following GPS-error) that can be set by the operator. Usually, data with a higher error are stored only if they are acceptable for the operator. These GPS-errors can be caused by: (a) a non-favorable satellite geometry, (b) the presence of some obstacles on the topographic surface (i.e., trees or mountains), or (c) low quality in the signal transmission.

All the presented papers discuss the influence of the electrodes’ position on the acquired and/or inverted soil resistivity data without taking into account the effects of errors occurring during a high-precision electrode positioning measurement campaign. The accuracy of a GPS system, for instance, can strongly affect the quality of coordinates used in an ERT inversion software, influencing soil resistivity output data. In this paper we present the first results of an ongoing project among three different departments of the University of Firenze: the Etruscology Chair of the Department of History, Archaeology, Geography, Fine and Performing Arts (SAGAS), the Department of Earth Sciences (DST), and the Department of Information Engineering (DINFO). The first research group (thanks to the authorization of the Soprintendenza Archeologia Belle Arti e Paesaggio for the Provinces of Arezzo, Siena and Grosseto) has decided to undertake a new excavation campaign on Poggio Pepe Etruscan tumulus, in Tuscany (Italy). Before that, a geophysical survey of the tumulus has been carried out by the DST research group to verify the state of conservation of buried structures and to identify the ancient access of the tomb. Thanks to the collaboration with the DINFO research group, an in-depth statistical analysis of the acquired data was carried out by means of a Monte Carlo (MC) simulation [35,36]. MC method was applied to evaluate the influence of the GPS-error on the definition of the geometric factor and consequently on the indirect measurement of the apparent resistivity. Therefore, the main research questions were: (i) if, during the inversion procedure, it is correct to “ignore” the effects of GPS-errors collected during the field survey (i.e., use the GPS values without performing a statistical analysis on the errors that affect the measure and correct the GPS values taking into account these errors), and (ii) if exist a minimum error threshold over which the inversion procedure is significantly affected by it. Optimizing the acquisition parameters (in terms of array and input voltage) and the inversion software input data (i.e., taking or not into account the GPS-error) it is possible to reconstruct a more reliable subsoil model and improve the anomalies location. Consequently, the archaeological excavation survey can be designed easily, the research funding can be managed more efficiently, and the excavation survey costs can be minimized.

In Section 2 the study area is initially presented, then the geophysical survey and the data elaboration procedure, and finally the MC method applied to the data set of interest. In Section 3 the results of both the geophysical survey and the MC simulations are shown, while in Section 4 an in-depth discussion is provided.

## 2. Materials and Methods

### 2.1. Study Area

**Figure 1:**
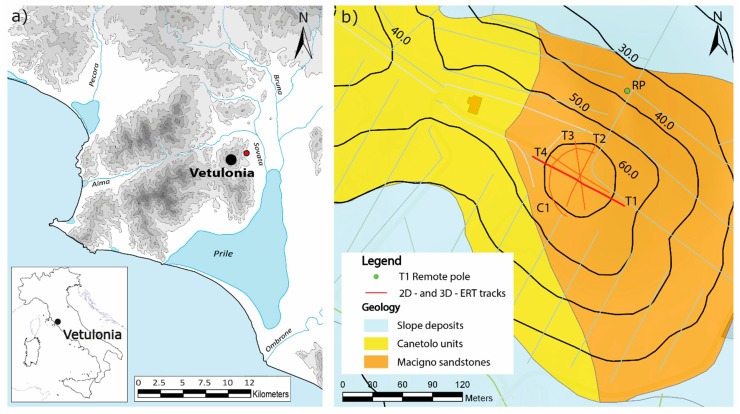
Maps of the study area. (**a**) Ancient map of the Etrurian region around Vetulonia in the 6th century BC, with the location of Poggio Pepe Tumulus (red dot). (**b**) Locations of the 2D-ERT (T1-T4, red lines) and 3D-ERT (C1, C-shaped red line) over the geological map of the study area. Light green dot (RP) represents the remote pole employed in the T1 pole-dipole acquisitions.

Vetulonia (Tuscany, central Italy, Figure 1a) was one of the most important Etruscan towns of 6th century BC and played an important role in the Etruscan League of twelves cities. Many monumental tumuli were erect by the powerful Vetulonia princes. These funeral mounds are among the largest in the ancient world, with diameters that can reach up to 90 m. Under the Roman Empire, Vetulonia shrank to a secondary center, but ancient remains of cyclopean walls and monumental necropolis testify its cultural and economic importance. At the end of 19th century AD, the archaeologist Isidoro Falchi excavated many funeral mounds in Vetulonia area and the relics discovered can be currently found in the museums of Grosseto and Firenze. Etruscan tombs consist of a long access corridor (known as dromos) and a quadrangular shaped funeral chamber, with one or more rooms on the two sides, reproducing the original house of the deceased’s family. Walls and roofs are built in earth and stones slabs, with several rock carved interiors. Most important mounds are generally covered by peculiar rounded tumuli that form an important feature in surrounding landscape. Poggio Pepe tumulus has a diameter of about 90 m and is the fifth largest tumulus known up to know in Tuscany. It is located North-East of Vetulonia town, facing toward the area where the ancient port of Vetulonian on the Prile lagoon was likely located (Figure 1a). It was firstly detected and investigated by Isidoro Falchi at the beginning of 1900 by means of some excavation probes that brought to the light the beginning of the dromos. Unfortunately, the evidence of previous looting discouraged further excavations [37,38]. Thus, the internal chambers of Poggio Pepe mound are still unexplored. Many large tumuli are characterized by a marked depression at their top, identifying the presence of a potential collapse that affects the roof of the main room. However, the Poggio Pepe tumulus does not show any morphologic deformation, suggesting that no one entered into the tomb from the roof, and therefore the internal structure of the tomb could be still intact.

From a geological point of view (Figure 1b), the area around Poggio Pepe tumulus is interested by the presence of Macigno unit sandstones (Upper Oligocene/Lower Miocene) and shales and limestones of the Canetolo unit (Paleocene/Eocene). Moreover, holocenic slope deposits with limited thickness can be found in the northern and southern parts of the area.

### 2.2. Geophysical Measurements: Field Survey and First Elaboration

Between the end of August and the first half of September 2019 two different field surveys were carried out at the test site with different purposes and therefore different electrode placement settings. The goal of the first survey was to evaluate the influence on the collected data, and thus on the inverted resistivity model, of the GPS-error and of the measured voltage errors [25]. Moreover, the archaeologists were interested in locating the tumulus dromos and the maximum depth of the resistive anomalies associated with the funeral chamber. Thus, to achieve this last aim, a C-shaped 3D-ERT (C1 in Figure 1) was performed, while for the first one, along a 2D profile (T1 in Figure 1), apparent resistivity data with both DD and PD configurations and different input voltage were collected.

Commonly speaking, the input is a “current”, thus in technical jargon the two input electrodes (named A and B) are called “current electrodes” [16], and the other two electrodes (named M and N) are called “voltage electrodes”. Actually, the input current is generated in the soil as a consequence of the different voltage applied by the instrument at the two current electrodes. A 10-channels receiver SyscalPro 48-electrodes by Iris (Orléans, France), joined with an external link (to reach a total amount of in-line 72 electrodes) were used to collect data. This instrument allows to set the input voltage (V_AB_) as a constant value (12 V, 25 V, 50 V, 100 V, 200 V, 400 V or 800 V) or to set a constant voltage (Vp) that has to be read at the M and N (20 mV—called Save Energy mode—, 50 mV, 200 mV, and 800 mV). This second set-up lets the instrument modify at each iteration the input voltage that could be at least equal to a maximum V_AB_ value forced by the operator. Of course, the imposed values are reached, thanks to the auto-ranging, only if the instruments limits (e.g., a maximum current of 2.5 A) are satisfied. For the DD array 5 different input were chosen (V_AB_ equal to 200 V, 400 V, and 800V and Vp equal to 800 mV and Save Energy with a V_AB-max_ equal to 800 V), why only two for the PD one (V_AB_ equal to 800V and Vp equal to Save Energy with a V_AB-max_ equal to 800 V). The data acquisition sequences were generated to take into account 10 and 5 increments of n and a, respectively, that are the distance between B and M and the fixed distance between two consecutive electrodes [3,27]. Therefore, each DD and PD ERT has 2475 and 2625 acquisitions, respectively. Thanks to the multi-channel system each ERT takes about 10–15 min. The acquisition parameters are summarized in Table 1.

The two selected arrays are quite often employed in archaeological surveys coupled or not with a multi-gradient array [17,19,22]. Moreover, they were chosen since they have a similar imaging resolution that is better than other arrays for locating vertical anomalies (like anthropogenic structures) at shallow depths [3,22,27]. Finally, given the purpose of the survey, these arrays were also chosen for their disadvantages that are: (i) a larger risk of noise contamination and (ii) a relatively low signal to noise ratio, if compared with other arrays [21,25,27]. DD is also more subject to in-line spacing errors [25] and topographic effects [28]. Both T1 and C1 had a theoretical inter electrode distance (a) equal to 1.5 m (linear length 106.5 m). The maximum investigation depth reached by the T1-DD and T1-PD configurations was of about 20 m and 30 m, respectively [39]. A Leica 1200 DGPS (Leica Geosystems S.p.A., Florence, Italy), that operates in Real Time Kinematic mode, was employed to collect the real electrode position. The acceptable GPS-error was set equal to 0.05 m.

**Figure 2:**
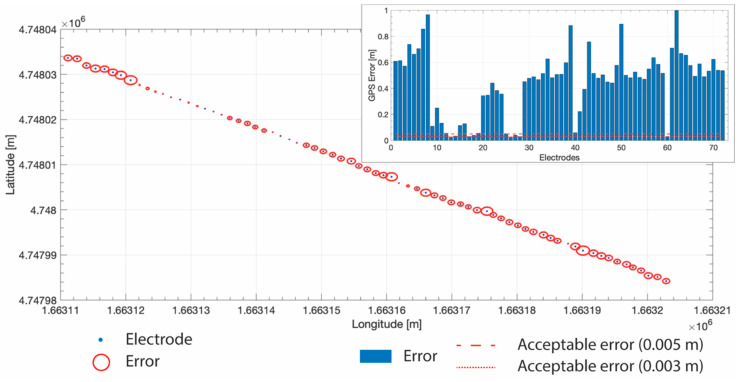
The GPS-errors, that affect the coordinates measures of each electrode, shown as blue bar (in the insert in the upper right corner) and as red circle around the “real” electrode position in the main panel (x and y axes are the longitude and latitude coordinates, respectively, in the Gauss Boaga-Roma40 projected system). In the insert (upper right corner) the red dashed line at 0.05 m is the imposed GPS threshold employed in the field acquisition, i.e., the error value imposed as acceptable (see text), while the red pointed line at 0.03 m is another possible threshold commonly used in field acquisition.

In GPS analysis field, quality of the acquired positioning data can be determined by examining the Dilution of Precision (DOP) parameters, such as Position Dilution of Precision (PDOP) and Horizontal Dilution of Precision (HDOP). The number of satellites available during a survey can also be an important indicator of data reliability. During the field survey, the presence of several obstacles in the study area (i.e., high trees and terrain geomorphology) limited the number of simultaneous available satellites (always lower than 6) and induced a non-favourable satellite geometry, and therefore high values of PDOP and HDOP have been encountered. In particular, for all acquired GPS data, average DOP values ranged between 7.3 and 9.5 for PDOP and between 8.8 and 10.2 for HDOP. Because of such poor satellite geometry, only the 11.0% of the T1 data were stored with an error lower than 0.05 m, and among this only the 2.8% has an error lower than 0.03 m (Figure 2). Even if it was not expected to acquire data with an error higher than the acceptable threshold, it did not damage the test. Nevertheless, given the high values of the errors only planimetric (xy) GPS-errors were taken into account to avoid unrealistic (saw-shaped) topography profiles and therefore abrupt changes in elevation that cold not been properly modelled by the resistivity inversion software and that odiously introduce unrealistic resistivity anomaly in the outputs.

The aims of the second field survey were to locate the funeral chamber, trying to verify the state of conservation of the roof, and to identify the ancient access to the tomb. Three radials [18,22] 2D-ERTs (T2-T4 in Figure 1) with a theoretical inter electrode distance equal to 1.0 m (linear length 71.0 m) and with DD and PD configuration were acquired. Given the spatial distribution of these ERT, and also according to the recent literature about radial ERTs on tumuli [18,22], C- or O-shaped 3D-ERTs on funeral mounds [17], and imaging resolution of 3D-ERT [26], it is obvious that more ERTs are necessary to cover the whole tumulus and better reconstruct the subsurface anomalies. Therefore, as mentioned also in the introduction, the results of C1, T2-T4 will not presented and discussed in this paper, while it is possible to find a brief presentation of the first results in Pazzi et al. [40].

To elaborate the acquired apparent resistivity data the commercial software ErtLab^TM^ was used [4,12,13,34]. It employs a finite element method that divides the subsoil model into triangular cells. Thus, it is more flexible with rough topography or structures [13,28]. The software is able to model a flat surface or take into account the actual electrode position. It also implements a finest data noise management, using the Occam’s regularization [41] as developed by Morelli and LaBrecque [31]. This allows to estimate and assume for the inversion different percentage values of the standard deviation noise, according to the quality of the dataset [12,13]. Finally, starting from a homogeneous resistivity half-space, it calculates the quality of an inversion minimizing the misfit function between the field and modelled data [4,13]. The starting apparent resistivity was chosen equal to 195 Ωm, i.e., equal to the mean values of the whole acquired and simulated dataset (see Section 2.4). Given the good quality of the acquired data, only few measures were removed from the DD and PD dataset (Table 1) on the basis of the standard deviation percentage value fixed lower than 2%. Moreover, the “data noise error” was set equal to 1%. Given the purposes of the present work, all the ERTs were elaborated both with (topo in the following) and without (flat in the following) taking into account the real geographic coordinates of the electrodes.

### 2.3. Monte Carlo (MC) Simulations to Take into Account the GPS-Error

The well-known apparent resistivity equations [3] link the measured electric parameters (current and voltage) with the mutual distances among the electrodes employed (geometric factor, k). Therefore, to assess the GPS-error influence on the indirect measurement of the apparent resistivity, the first step was to evaluate the influence on the geometric factor. This evaluation was performed by means of a MC simulation, that is essentially defined as a problem-solving technique. It is commonly used, in fact, to obtain the probability approximation and the statistical characteristics of the results (model outputs) by testing multiple trial runs, called simulations, relying on variables randomly selected [36,42,43,44]. The data analysis procedure, carried out for the purposes of the work, can be summarized in the following seven steps: (1) acquisition of the MC simulation input data, i.e., voltage at the MN dipole, current at the A and B electrodes, and absolute (geographical coordinates) and relative (coordinates referred to the first electrode) position of the electrodes); (2) construction of a electrodes possible location set taking into account for each electrode its uncertainty given by the GPS; (3) setting at 100,000 the number (N) of MC iterations; (4) generation of a random input (A, B, M, and N electrodes location) over the domain according to a chosen probability distribution (uniform and normal); (5) calculation, using the values generated at step 4, of the geometric factor (k) and of the apparent resistivity (ρ_a_) for each quadripoles of the acquired sequence; (6) repetition of the steps 4 and 5 N times; and (7) collection of the final results.

As well-known, the two probability distributions, chosen at step 4, are both continuous and have the same mean. The uniform one is symmetric, i.e., all the values in the distribution interval are equally probable, and the range is defined by the minimum and maximum of the possible location point [35]. Considering X the measured value and ε its GPS-error, the admissible range of values is [X − ε, X + ε]. The normal or Gaussian distribution, at the contrary, has a single central peak at the mean value and is described by a bell-shaped curve with the graph falling off evenly on either side of the mean [35]. It has been calculated taking into account 3σ = ε (where σ is the standard deviation) i.e., that the 99.73% of values lie within the same range used for the uniform distribution.

To evaluate the influence of considering GPS-error in generating apparent resistivity data, the error in the geometric factor (δk_flat_ and δk_topo_, respectively) was calculated, for both the flat and topo models, defined as follows (see also: [29,30]):

(1) $${\delta k}_{\mathrm{flat}}=100|\frac{k_{\mathrm{GPSflat}}-k_{\mathrm{theoflat}}}{k_{\mathrm{theoflat}}}|$$

(2) $${\delta k}_{\mathrm{topo}}=100|\frac{k_{\mathrm{GPStopo}}-k_{\mathrm{theotopo}}}{k_{\mathrm{theotopo}}}|$$

where k_GPSflat_ and k_GPStopo_ are the geometric factor calculated at step 5 considering, at step 4, relative (flat) or absolute (topo) values of the electrodes coordinates, k_theoflat_ is the geometric factor obtained considering a flat surface and a fixed electrode distance equal to a = 1.5 m, and k_theotopo_ is the geometric factor computed taking into account the real electrode location (the collected GPS values).

### 2.4. MC Simulated ERT Comparison

The MC simulation of each acquired dataset allows to obtain “simulated” apparent resistivity dataset that were further employed in the comparison analysis. In particular, dataset with values equal to the mean, the median, the 1st, 25th, 75th, and 99th percentiles of the whole 100,000 simulations were taken into account. Minimum, maximum, and mode dataset were also generated, but not employed. In fact, minimum and maximum dataset enclose only MC simulation outliers, and therefore could provide unreliable outcomes. Considering that the mode is the value that appears most often in a data set, in this case the mode is a meaningless parameter since the results of each simulation are real number always different from each other, and therefore there are not repeated values. The simulated datasets were thus inverted by means of ERTLab software with the same configuration employed to elaborate the field dataset (i.e., removal of data with a standard deviation higher than 2%, a starting apparent resistivity equal to 195 Ωm, and a data noise error equal to 1%). Data collected during the field campaign that do not take into account the GPS-error, and therefore their inverted model, are called “original” from now on, while all the others, that take into account the GPS-error, are called “simulated”.

In the ERT field, the anomaly effect (AE) has been introduced, since the end of the ’70s, to estimate the array effectiveness and imaging capability [7,26,27,29]. This parameter is a measure of the signal to noise ratio (SNR), because the signal has to be appreciably greater than the background noise [26]. Given an electrode configuration, the AE value depends on the electrical anisotropy subsoil distribution. Therefore, in this paper this parameter has been introduced to assess the influence of the GPS-error on the apparent resistivity. In fact, if the AE values of the original and simulated dataset are comparable, it means that the simulated datasets, that take into account the GPS-error, are not so different from the original one, and therefore, that the GPS-errors are not so influent. At the contrary, if the simulated AE values are significantly different from the original AE, it means that resistive anomalies or artefacts in the simulated dataset are generated by the GPS-error. In particular, two different AE were defined according to Equations (3) and (4): one “general” (mean absolute AE, Equation (3)), using the same definition of [26], that consider that the parameter is a measure of the whole inverted model quality, and one “level” (lAE, Equation (4)), according to [29], that take into account the different depth of the information provided (i.e., level by level). Considering that the increments of the inter-electrode distance (a) varies in the range [1,2,3,4,5] and the increase of the distance B-M (n) varies in the range of [1,2,3,4,5,6,7,8,9,10], we obtained 44 and 41 levels for the DD and PD array, respectively. AE and lAE are defined as follow:

(3) $$\mathrm{AE}=\frac{\max\left(\rho_{a}\right)-\min\left(\rho_{a}\right)}{\mathrm{mean}\left(\rho_{a}\right)}$$

(4) $$\mathrm{lAE}=\frac{\max\left(\rho_{a}\right)-\min\left(\rho_{a}\right)}{\mathrm{mean}\left(\rho_{\mathrm{alevel}}\right)}$$

where, for each dataset, max and min are the maximum and minimum values of the whole apparent resistivity values (ρ_a_), while mean is the average of the whole apparent resistivity values (ρ_a_) or of the apparent resistivity at a given level/depth (ρ_alevel_), that means for each combination of (a) and (n).

Consistently with Razafindratsima and Lataste [29], to compare simulated (ρ_simm_) or not (ρ_orig_) resistivity inverted models the ERT-error (δERT) was defined as follow (Equation (5)):

(5) $$\delta \mathrm{ERT}=\delta \rho =100|\frac{\rho_{\mathrm{simm}}-\rho_{\mathrm{orig}}}{\rho_{\mathrm{orig}}}|$$

Unfortunately, since the differences between the simulated and not simulated datasets are very high, δERT has values higher than 100. Therefore, to better compare ERT-error results a normalized value, that range between 0 and 1, was introduced as follows:

(6) $${\delta \mathrm{ERT}}_{\mathrm{norm}}={\delta \rho }_{\mathrm{norm}}=\frac{\delta \mathrm{ERT}}{{\delta \mathrm{ERT}}_{\max}}$$

where δERT_max_ is the maximum value of the simulated dataset for each different voltage input.

**Figure 3:**
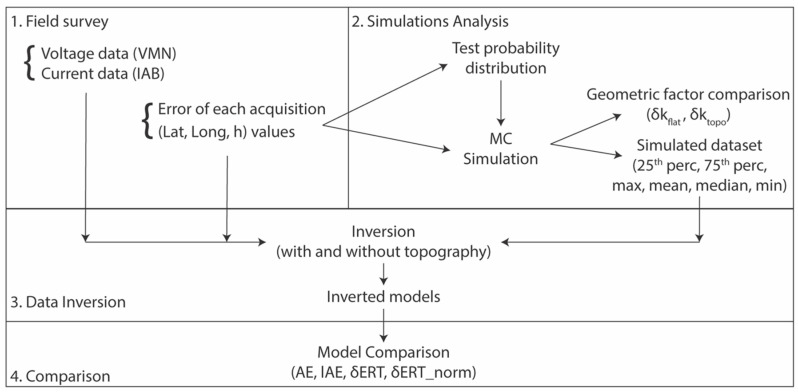
Flow chart of the whole procedure. Four main steps can be identified: 1. The field data acquisition of both electrical soil parameters (“original” data) and geographical coordinates of the electrodes, 2. The MC simulation to take into account the GPS-errors, 3. The “original” and simulated electrical resistivity data inversion with (topo) and without (flat) topography, 4. The models comparison to evaluate the GPS-error influence.

The flow chart of the whole procedure is illustrated in Figure 3.

## 3. Results

This section is divided into three subsections. In the first one, the main results, from an archaeological point of view are shown, in the second the results of the MC simulations are presented, while in the third their comparison with the original inverted model/dataset is illustrated.

### 3.1. Survey Results

**Figure 4:**
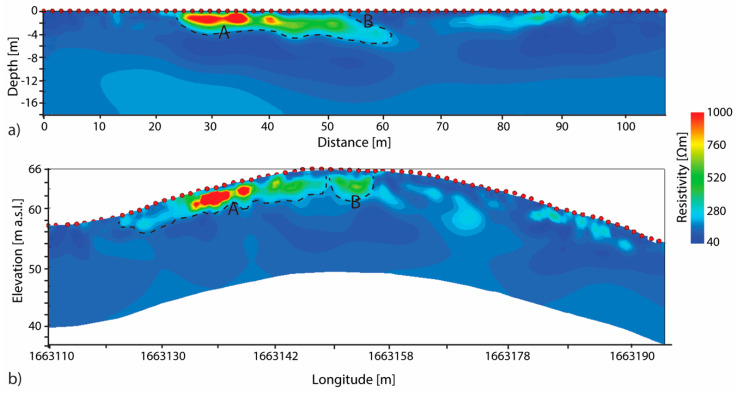
T1-ERT resistivity profiles obtained inverting at the same time DD and PD acquired data. (**a**) ERT profile obtained without take into account the topography (flat) and (**b**) taking it into account. Anomaly A and B can be associate to the dromos rocky blocks and the shadow of the funeral chamber, respectively.

The study area is characterized by resistivity values lower than 250 Ωm (blue colours in Figure 4), typical for sandstone that, according to the geological map (Figure 1), outcrops in the area. Both flat and topo resistivity sections (Figure 4a,b, respectively), obtained inverting at the same time DD and PD acquired data, show that between 22.5 m and 60 m up to a depth of 6 m there is a high resistivity anomaly (values higher than 500 Ωm associated to green and red colours). According to the archaeologist anomaly A can be associated to the rocky blocks used to build the anthropic structures. In particular, because its elongated shape, resistivities higher than 750 Ωm can be associated to the *dromos* and those in the range of 300 Ωm–750 Ωm to its shadow. ERT T1, in fact, was drawn to cross the hypnotized location of the *dromos*. Anomaly B can be associated to the shadow of chamber filled whether with soil or air.

### 3.2. MC Simulations Results

**Figure 5:**
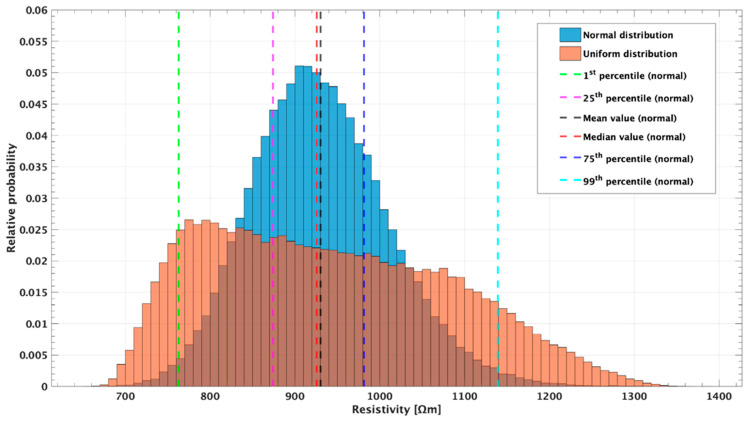
Histograms, for a randomly selected quadripole (the number 759 with A = 15, B = 17, M = 21, and N = 23, acquired with an input voltage of V_AB_ = 800 V), of the apparent resistivity values obtained by the MC simulation employing 100,000 different positions of the electrodes selected using both uniform (orange) and normal (blue) distributions. For the normal distribution, values of 1st percentile (green dashed line), 25th percentile (pink line), mean (black line), median (red line), 75th percentile (blue line), and 99th percentile (light blue line) are reported. The 759 quadripole apparent resistivity is 926.625 Ωm.

As described in Section 2.3, each of the 100,000 MC simulation is carried out taking into account different electrodes positions. These positions are randomly selected, according to a normal or uniform distribution, from a domain of possible values calculated on the basis of the uncertainty given by the GPS for that electrode. Therefore, for each quadripole is possible to calculate the distribution of the apparent resistivity obtained in all the 100,000 MC simulations. Figure 5 shows this resistivity distributions of a randomly selected quadripole (i.e., the number 759 with A = 15, B = 17, M = 21, and N = 23, acquired with an input voltage of V_AB_ = 800 V). These two distributions were obtained extracting the positions of A, B, M, and N according to a uniform (in orange in the figure) and a normal (in blue in the figure) distributions (see Section 2.3).

The two histograms highlight a great variability of the apparent resistivity when the localization of the electrodes is affected by errors. In particular, the uniform distribution range seems to be larger than that of the normal one. Indeed, the uniform range (667.10–1365.00) is quite smaller than the normal one (651.21–1386.80). This is because the normal distribution tends to infinite, and therefore has isolated values at the extremes. The normal distribution, compared to the uniform one, better maintains its shape. The colored dotted vertical lines represent the apparent resistivity values of considered statistical parameter for the normal distribution. These values, together with that for the uniform distribution, are summarized in Table 2.

**Figure 6:**
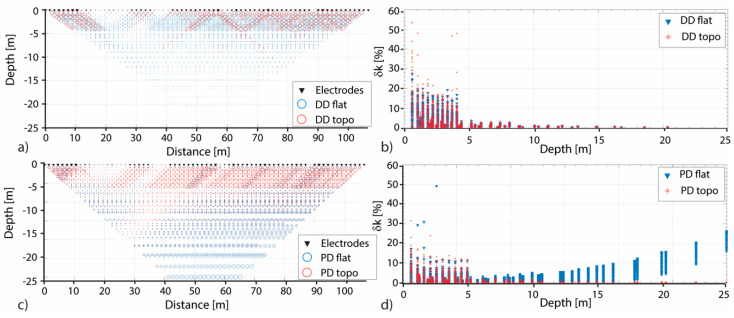
Distributions for the DD and PD arrays of the errors in the geometric factor (δk). In (**a**) and (**c**) for each quadripole the δk is the radius of a circle, while in (**b**) and (**d**) the δk numerical values are plotted versus the depth. In all the panels blue represents the δk values for the flat solution, while red that for the topography one.

The influence of the GPS-errors on the geometric factor errors (δk_flat_ and δk_topo_ for both the DD and PD arrays is show in Figure 6. It is possible to observe that, up to a depth of 5 m, δk_flat_ and δk_topo_ strongly suffer the electrodes mispositioning effect. These results confirm what known from the literature, i.e., that the DD percentage error is higher (quite double) than that of the PD array [14,25]. It is also important to note that considering a flat topography, at depth higher than 7 m, the PD error is no more influenced by the GPS-error, but it increases with the depth [29].

### 3.3. Simulated ERT Results

**Figure 7:**
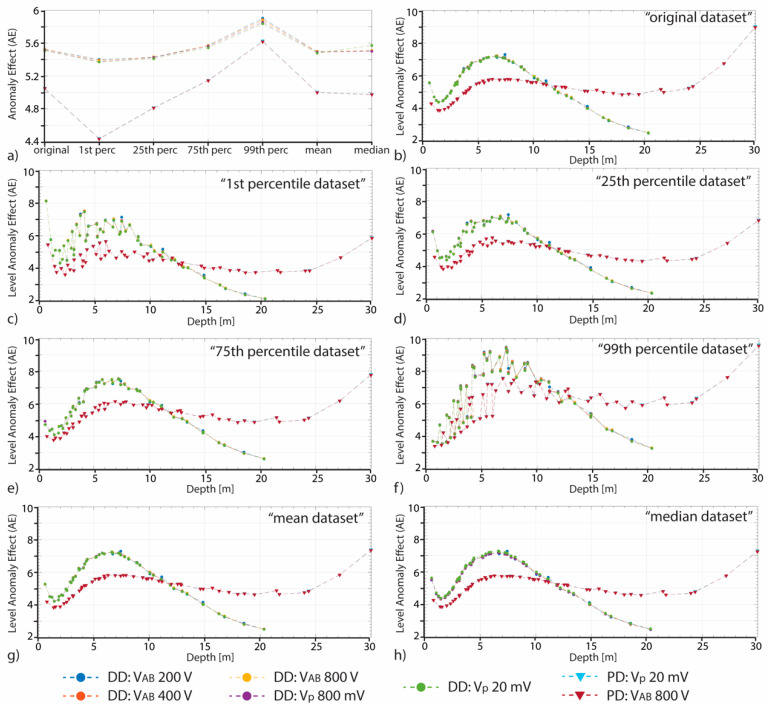
(**a**) The DD (marked with dots) and PD (marked with triangles) global anomaly effect (AE) for each original and simulated dataset. (**b**–**h**) The DD and PD level anomaly effect (lAE) for the original and simulated (1st percentile, 25th percentile, 75th percentile, 99th percentile, mean, and median) datasets.

The AE and lAE values of each original and simulated dataset, calculated according to Equations (3) and (4), respectively, are shown in Figure 7 for both DD and PD arrays. It is possible to note that the DD-AE values are higher with lower spread than the PD-AE ones. Moreover, the PD-AE’s variability is higher (almost 1 point from 4.432 of the “1st percentile” dataset up to 5.629 of the “99th percentile” dataset). For both the arrays, independently of the input voltage, the AE maximum values are those of the 99th percentile datasets, while the minimum values are those of the 1st percentile datasets. The lAE values variability is higher than that of the AE. In particular, the highest values are reached by the PD-lAE at the maximum investigation depth, and in general the maximum lAE values are those of the 99th percentile dataset for both the analyzed arrays. The mean and median lAE trends are quite similar to the original one, while the others are more spreaded, especially in the first 10 m depth. The DD-lAE minimum values are reached in the last level (maximum depth of investigation), while that of the PD array are observed in the fourth level (at 1.45 and 1.97 m for the DD and PD, respectively). In general, the DD-lAE curves have a minimum at the fourth level, then increase up to the 31st level (7.3 m depth, with a tangent in the range [0.467–0.509]) reaching a relative maximum, and then decrease again quite slower (the tangent is in the range [0.353–0.373]). The PD-lAE curve trend is comparable in the first 7 m depth (the relative minimum is reached at the third level), while up to 20–25 m depth it is quite stable, and then increase faster. These results suggest that the 1st percentile and 99th percentile simulated dataset are those more affected by the GPS-error.

**Figure 8:**
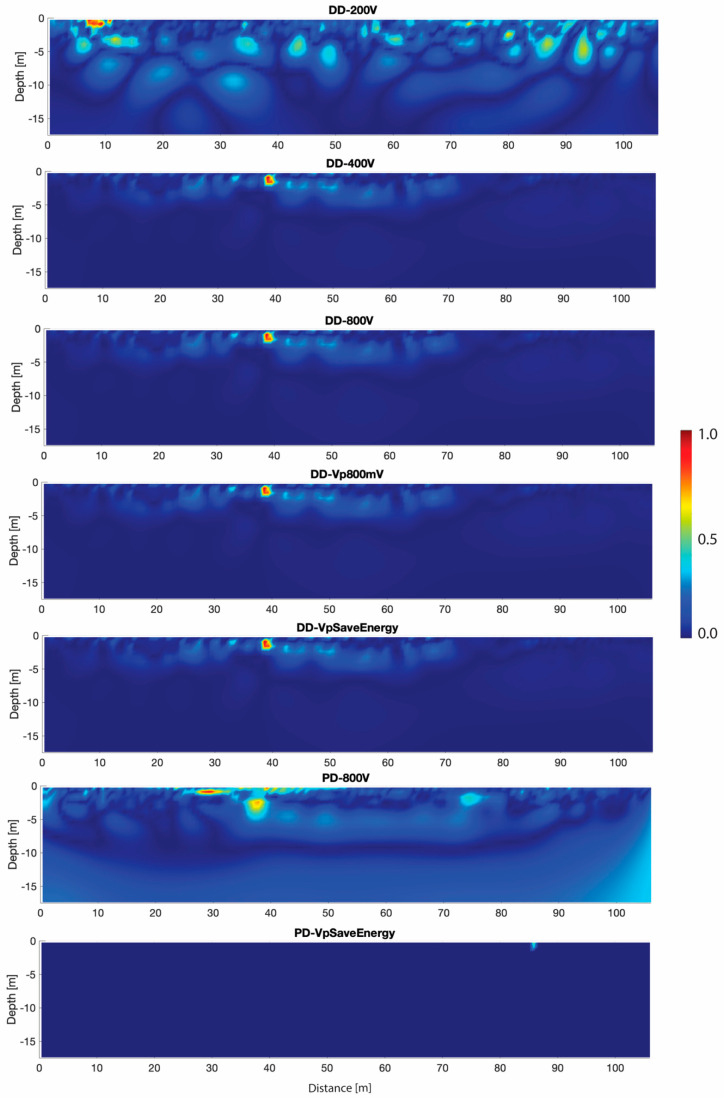
Normalized ERT-error (δERT_norm_), for each input voltage, of the mean simulated dataset.

The normalized ERT-error (δERT_norm_, see Equations (5) and (6)) of the mean simulated datasets, obtained for each input voltage, is shown in Figure 8. For the DD array, apart for the input voltage equal to 200 V, the highest errors of the inverted models are located within the first 5 m of depth from the ground surface and at a distance of [35 m–40 m] in correspondence of a high resistivity anomaly. The normalized ERT-errors of each simulated dataset for the 200 V input and the PD δERT_norm_ show the highest variability.

## 4. Discussion

A misplacement of the electrodes in an ERT acquisition could generate errors in the array geometric factor and a distortion of the measured potential field in the ground. Therefore, if the real electrode position is not taken into account in the inversion procedure, and indeed the electrode theoretical position is used, the artifacts caused by the wrong input data can be interpreted as anomalous resistivity of interest.

In the present study, an ad hoc ERT field survey was conducted (see Section 2.2) and apparent resistivity data, together with GPS data of electrodes position, were collected at the Poggio Pepe archaeological site. Thus, by means of a MC simulation, six different simulated datasets were generated for each acquired dataset (considering 1st, 25th, 75th, 99th, mean, and median statistical indexes). To generate random inputs over the domain of possible electrodes location, both the uniform and the normal distributions were tested. Both have the same relative error trend (Figure 5), but the normal distribution is characterized by an average error approximately three times lower than the other. Thus, results suggest that the normal distribution is more suitable and reliable to approximate this type of uncertain data. This result is in agreement with that of Clement and Moreau [14] who found that, at a laboratory scale, the electrode-spacing error follows a normal distribution. Thus, this probability distribution was chosen for all the MC simulations. To evaluate the influence of the GPS-error on the acquired and inverted data, four different parameters were defined and analyzed: the error in the geometric factor (δk), the global (AE) and level (lAE) anomaly effects, and the normalized ERT-error (δERT_norm_).

Plotting the δk_flat_ and δk_topo_ values against with/respect to the investigation depth for both the DD and PD arrays (Figure 6) it is possible to observe that, within the first 5 m, geometric factor distribution is influenced by the GPS-error. Moreover, the PD measurements are also affected by a greater error at a depth deeper than 7 m (Figure 6). These results are in agreement with observations of Razafindratsima and Lataste [29]. The trend of this error, in fact, is comparable to that of the error induced by considering a finite position of the remote pole instead of an infinite theoretical one. Therefore, this result confirm, as known from literature, that (a) at shallow depth, the DD array is more influenced by the GPS-error than the PD array and (b) introducing the topography in the inversion procedure, it reduces the error in depth generated by the finite position of the remote pole, that seems to have an exponential trend. It is also possible to find in the literature that the geometric error sensitivity could be used to remove measurements more sensitive to the electrode-position error [15,23]. Nevertheless, this function is quite complicate. At the contrary, the proposed parameter δk can be easily used. Considering that archaeologists are often interested in investigating the first few meters of the underground, δk can be employed to evaluate which is the GPS-error influence on the acquired dataset and to decide which quadripoles/measurements are more affected by the GPS-error and have to be removed. Given the purposes of the presented work, no measurements were removed from the datasets, and all the apparent resistivity values were taken into account in the inversion procedure.

As discussed in the literature, the AE value of a given electrode configuration depends on the electrical anisotropy subsoil distribution [7,26,27,29]. Therefore, in this study this parameter has been employed as an indicator of the differences generated in the apparent resistivity dataset by the GPS-error, assuming that higher the AE value, higher the generated apparent resistivity artefacts are. The AE values obtained in the present study show bigger values than those obtained by Razafindratsima and Lataste [29] but quite similar to those of [26]. This is probably caused by the range of apparent resistivity, that, compared to the first one, is three orders higher, while is similar in magnitude to the second one. Figure 7 and Table 3 show that, for each simulated and original dataset, the DD-AE is higher than the PD-AE. This result, considering only the “original” dataset, is in agreement with the known, higher capability of the DD array to better capture the subsoil resistivity anomalies [27]. Taking into account the values of the simulated dataset, it is possible to note that the AE of the mean and median dataset are quite similar to the “original” one (Table 3). Therefore, it indicates, as expected, that these two datasets are not so influenced by the GPS-error. At the contrary, the AEs of the 1st percentile and 99th percentile datasets indicate a higher influence of the GPS-error, in agreement with the definition itself of these simulated datasets. In fact, these two datasets are those that enclose 1% and 99% of the 100,000 MC simulations, respectively, that means that also extreme values are considered. The influence of the GPS-error on these two datasets is also testified by the highest number of the iterations (Table 4) needed to reach the model convergence. 1st percentile and 99th percentile datasets, in fact, are characterized by a wide range of apparent resistivity data, and the differences among values are higher. Therefore, the software needs more iterations to converge. Finally, observing the higher PD-AEs spread (almost 1 point, as observed in the result section and visible in Table 3), it is possible to assess that the PD array is more influenced by the GPS-error. These results are in agreement with that about the δk.

**Figure 9:**
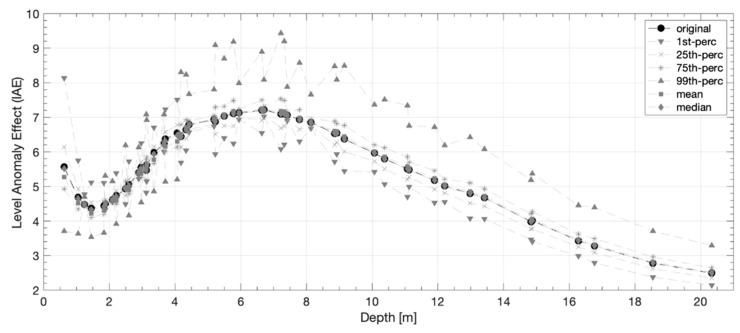
The DD and PD level anomaly effect (lAE) for the original and simulated (25th percentile, 75th percentile, maximum, mean, median, and minimum) datasets obtained with an input voltage of 200 V.

The AE is an average value of the whole dataset. Observing the trend of the lAE for each dataset considering different input voltages (Figure 7) and the trends of the lAE values of the different dataset of a chosen input voltage (Figure 9), it is possible to note that the influence of the GPS is not uniform, but it varies within the array (i.e., it depends on the depth). In particular, independently on the GPS-error, the smaller variations of the lAE at high depth (Figure 7 and Figure 9) could be related to the lower number of the acquired data (i.e., the array definition decrease with depth). This result is true only for the DD array. The PD array, in fact, as already said analyzing the AE and lAE values, and as reported in literature [29], is more influenced (e.g., higher values of the lAE) at higher depth. Analyzing the lAE trends, for both the DD and PD arrays, it is also possible to observe that up to 11 m depth the DD-lAE is higher than the PD-lAE. This result is in agreement with the previous discussed higher capability of the DD array to better identify the subsoil resistivity anomalies. Furthermore, observing the lAE’s trends, it is possible to observe that the mean and median values are quite similar to the original one. Higher the variability among the simulated datasets, higher the influence of the GPS-error. All these considerations allow to assess that in presence of a flat topography it is better to take into account the GPS-error in the inversion procedure, using at least the mean MC simulated dataset.

**Figure 10:**
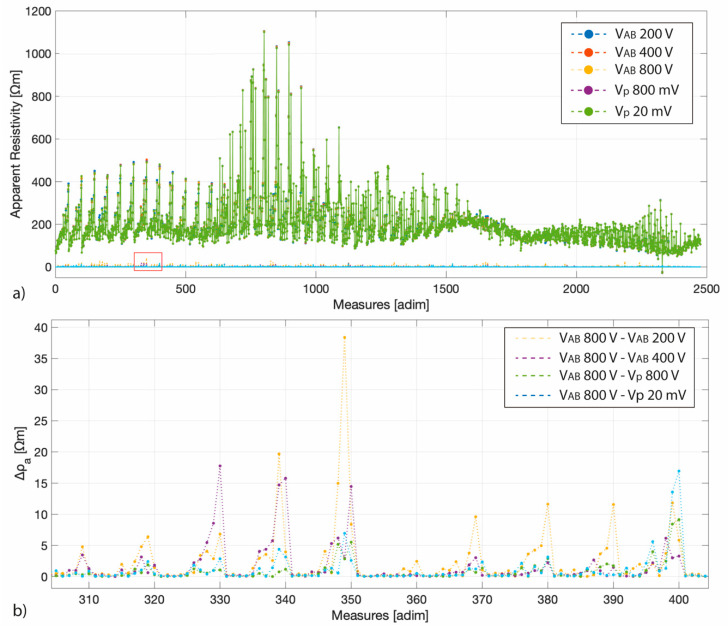
(**a**) The apparent resistivity values collected for each different input voltage and, around zero, the differences between the most energetic input (that with an AB voltage of 800 V) and the others. (**b**) A zoom on the differences of the measures between 305 and 405 (those highlighted by the red rectangle in panel (**a**)).

As for the AE, the lAE is not influenced by the input voltage, apart that of 1st percentile and 99th percentile datasets, that are those with the highest variability. The same consideration can be also demonstrated comparing the trend of the “original” apparent resistivity for the different employed input voltages (Figure 10) and the differences (Δρ_a_) between the most energetic input (that with an AB voltage of 800 V) and the others. Apart for some quadripoles, as highlighted in the Δρ_a_ zoom (Figure 10), the Δρ_a_ is around zero.

Figure 10 shows the results for the DD array, but the same results were obtained for the PD array. Therefore, considerations drawn on the basis of Figure 9 could be obtained also considering a different input voltage. 

It is important to note that the independence of the GPS-error influence from the input voltage is not an absolute result, but it is closely related to the case study, and in particular to its geological characteristics. In the presented case study, the subsoil conductivity distribution is quite homogenous and also a low voltage input (200 V or Vp Save Energy) allows to collect a high quality dataset (as mentioned in Section 2.2 fixing at 2% the standard deviation percentage few measures were removed from the DD and PD dataset, and the “data noise error” for the inversion procedure was set equal to 1%). Thus, it was not possible to evaluate if the GPS-error can be compensated changing the voltage of the input signal.

**Figure 11:**
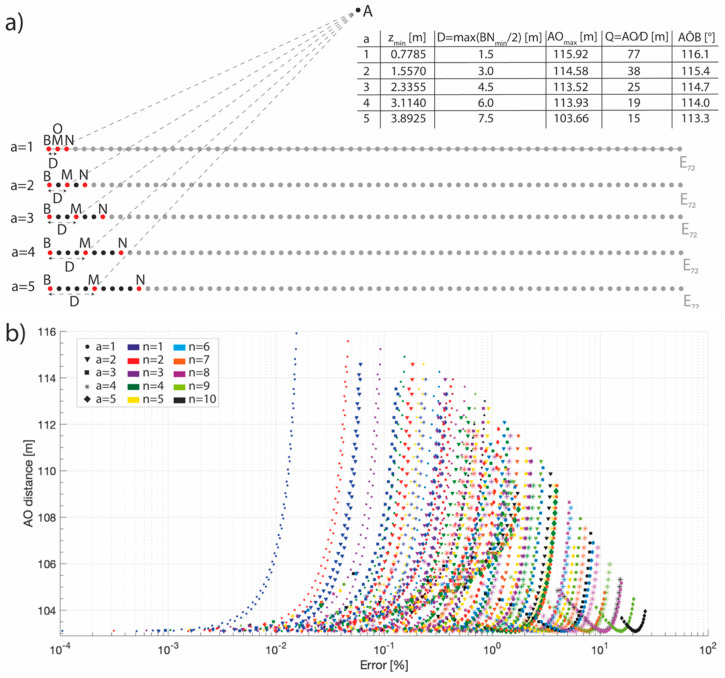
(**a**) Graphical description of the quantities involved in the computation of the infinite length coefficient (Q) and of the angle between the remote pole (A) and the first electrode of the tomography (B), calculated for each increment of the fixed inter electrode distance a according to the definitions provided by Razafindratsima and Lataste [29]. A and B are the current electrodes, M and N the voltage electrodes, O is the center of the maximum quadripole, and D the maximum quadripole half length. In the table, for each increment of a, are indicated the maximum investigation depth (after [39]), the D (the half of BN_max_ length) and AO lengths, and the values of Q and AÔB. (**b**) Distribution of the error, induced by the finite remote pole position, with respect to the AO length for each increment of the inter electrode distance (different symbols) and of the n value (different colours).

The ERTLab software, employed for the data inversion, is able to manage the electrode real positions, and therefore avoid artefacts caused by an electrode misplacement. To exclude that ERT-errors in PD acquisitions were also caused by the remote pole finite location, for each values of the fixed electrode distance (a), the infinite length coefficient (Q) and the angle (AÔB) between the remote pole (A), the centre of the maximum acquired array (O), and the first electrode (B) have been calculated [29]. The coefficient Q was firstly introduced by Robain et al. [30], discussing the influence of the remote electrode’s finite location in a pole-pole array. This coefficient is calculated as the ratio between the distance from the remote pole (A) to the centre (O) of the maximum acquired array (i.e., that with the current electrode B and the voltage electrode N_max_) and the half of the length of the maximum acquired array (D). They considered the maximum quadripole according to their remote pole position, i.e., in the first half of the electrodes line. A graphical description of the involved quantities is presented and values of Q and AÔB are summarized in Figure 11. Given the position of the remote pole, the maximum values of the AO distances were obtained for each values of the increment of (a), considering quadripoles with the minimum length. Because of graphical clarity, each increment of (a), belonging to the same ERT line, has been drawn on a different row. Consistently with the results of Razafindratsima and Lataste [29], if Q is in the range [2,3,4,5] and AÔB is about 100°, the influence of the finite position of the remote pole is not so relevant. Moreover, higher the value of Q, lower the influence. Taking into account the obtained values, it is possible to exclude an influence of the finite remote pole position on the inverted data, and therefore it is possible to assess that the observed ERT-error in the PD acquisitions is caused only by the GPS-error.

This result is also confirmed observing that the normalized ERT-error (δERT_norm_, Figure 8) of the PD acquisitions is comparable to that of the DD ones, evidencing the absence of the influence of the finite remote pole position on the dataset. Furthermore, the hypothesis that the DD is able to better capture the anomalous resistivity values is confirmed considering that, for each simulated dataset, the highest DD-δERT_norm_ is located within the first 5 m from the ground surface and in particular at a distance between 35 m and 40 m, in correspondence of the high resistivity anomaly interpreted by the archaeologist as a portion of the *dromos* (see Section 3.1). It can be assessed with a certain degree of confidence that this δERT_norm_ is generated by the anomaly itself and not by the GPS-error, because the electrodes over the anomaly are affected by a very low GPS-error (Figure 2 and Figure 6).

Zhou and Dahlin [25], in fact, demonstrated that the inverted model is affected by apparent artefacts just below the electrodes whose positions were affected by a 10% electrode-spacing errors. Finally, from a qualitative point of view, the ERT models (omitted here for brevity), obtained with the different simulated datasets, are comparable. The GPS induced errors, in fact, cause a resistivity variation compatible with the natural resistivity variability that do not influence the inverted model interpretation. Therefore, even if it is always better to consider the influence of the GPS-errors, both with a flat or a rough surface, by calculating the apparent resistivity with a MC simulation, the GPS-error influence is significant only from a numerical point of view.

**Figure 12:**
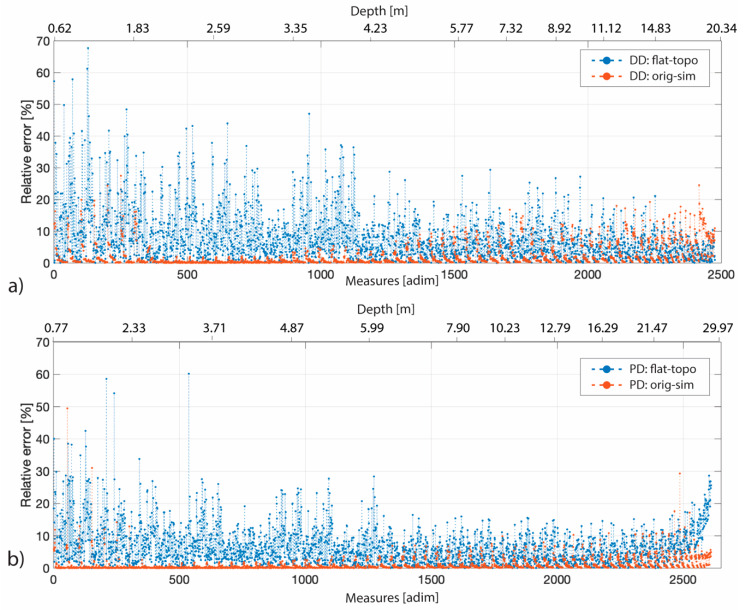
Geometric factor percentage relative error—with respect to both the depth (upper axis) and the number of measures ordered according to the quadripole investigation depth (lower axis)—between the flat and topo dataset (in blue), and between the original and the mean simulated datasets (in orange). (**a**) Both trends refer to the DD array while in (**b**) to the PD one.

The results on the analysis of the δkflat and δktopo show that in general the errors induced by the GPS on both the models, with and without topography, are lower than that introduced considering a flat subsoil model. This result is summarized in Figure 12, where for both the DD and PD arrays, the relative error induced if the topography is not taken into account is shown in blue, and that induced by skipping the GPS-errors in orange.

It is well known that z errors are usually higher than planimetric (xy) ones. Given the very low quality of our GPS dataset we preferred to not add also the z GPS-error to avoid unrealistic (saw-shaped) topography profiles. In fact, the software employed for the inversion models abrupt changes in elevation by smooth variations. We reserve to discuss the influence of the z GPS-error in a further publication employing data from surveys T2–T4 characterized by a lower GPS-error. Nevertheless, all the above considerations are confirmed if the topography and the planimetric GPS-error is taken into account, but for brevity these results are not presented here.

## 5. Conclusions

The main goal of this work was to evaluate, by means of a MC simulation, the influence of the GPS-errors that occur in collecting the geographical coordinates of the electrodes during an ERT survey, on the measured apparent resistivity data, and therefore on the electrical resistivity model. Firstly, an ad hoc field survey was conducted, and field data collected. For MC simulations, a probability distribution needs to be defined in order to generate the random inputs over the domain of possible electrodes’ location.

Both the uniform and normal distributions were tested, and the best and more reliable results were obtained thanks to the second one. For each acquired dataset, six different simulated outputs were obtained. To evaluate their quality, some reliability indexes were defined, like the geometric factor error (δk), the normalized ERT-error (δERT_norm_), the global (AE) and level (lAE) anomaly effects. The results of the analysis suggest that: (i) the GPS-error influence the apparent resistivity variations within the array; (ii) the PD array is more affected by the GPS-error, especially at higher depth; (iii) within the first 5 m the GPS-error influence is higher, compared with that observed at a depth between 5 m and 25 m; (iv) given the homogeneous conductivity distribution of the soil at the Poggio Pepe site, also a low voltage input (V_AB_ of 200 V of Vp Save Energy) was sufficient to collect a good quality dataset not strongly affected by the GPS-error, therefore, in this study it was not possible to define if the input voltage can help to compensate the GPS-error and this potential relation must be further investigated, (v) the GPS-errors induce artifacts of a lower amplitude compared with that generated by considering a flat model instead of one with the real topography. Therefore, even if the influence on the inverted model is not so relevant from a qualitative point of view, it is not possible to exclude the influence of the GPS-errors on the apparent resistivity and therefore on the inverted resistivity model. In case of archaeological application, where the area of interest is limited to the first meters underground, errors in collecting the GPS electrodes coordinates can generate anomalies that cannot be ignored, especially in presence of a flat topography.

## Figures and Tables

**Table 1:** Summary of the acquisition carried out along the T1 profile (see Figure 1 for the location) to test the influence of the GPS-error, occurred in collecting the electrode position, on different array (dipole-dipole: DD and pole-dipole: PD) and different input voltage.

Name	Array	Input	a	n	Removed Data	Iterations
T1-DD-200V	DD	V_AB_ 200 V	1–5	1–10	32	5
T1-DD-400V	DD	V_AB_ 400 V	1–5	1–10	4	6
T1-DD-800V	DD	V_AB_ 800 V	1–5	1–10	3	6
T1-DD-Vp800mV	DD	Vp 800 mV (V_AB_ max 800 V)	1–5	1–10	3	6
T1-DD-Vpsaven	DD	Vp 20 mV—SaveEnergy (V_AB_ max 800 V)	1–5	1–10	2	6
T1-PD-800V	PD	V_AB_ 800V	1–5	1–10	2	4
T1-PD-Vpsaven	PD	Vp 20 mV—SaveEnergy (V_AB_ max 800 V)	1–5	1–10	0	4

V_AB_ is the value of the input voltage at the current electrodes set constant and equal to 200 V, 400 V, and 800 V. Vp is the values that was imposed to be read at the potential electrodes (see the text for more details) set equal to 800 mV or 20 mV (Save Energy mode). a is the distance between two adjacent electrodes and n is the distance between B and M. The range values show the number of increments of these distances. The last column indicates the number of iterations necessary to reach the iteration procedure convergence.

**Table 2:** Statistical parameters of the two tested distributions (normal and uniform) for the randomly selected quadripole 759 (A:15; B:17; M:21; N:23) acquired with an input voltage of V_AB_ = 800 V which apparent resistivity is 926.625 Ωm.

Parameter	Normal Distribution [Ωm]	Uniform Distribution [Ωm]
1st percentile	762.95	705.21
25th percentile	874.09	820.98
Median	925.53	926.78
Mean	930.29	940.43
75th percentile	981.32	1048.90
99th percentile	1139.00	1235.20

**Table 3:** DD-AE and PD-AE values of the “original” and simulated datasets.

		Original	1st perc	25th perc	75th perc	99th perc	Mean	Median
DD-AE	200 V	5.524	5.401	5.427	5.572	5.904	5.495	5.507
	400 V	5.522	5.392	5.424	5.569	5.886	5.493	5.504
	800 V	5.528	5.384	5.434	5.572	5.877	5.500	5.510
	Vp800 mV	5.517	5.371	5.424	5.561	5.854	5.490	5.500
	VpSaven	5.507	5.370	5.410	5.544	5.836	5.479	5.572
PD-AE	800 V	5.051	4.438	4.815	5.148	5.629	5.004	4.977
	VpSaven	5.046	4.432	4.809	5.143	5.616	4.998	4.972

**Table 4:** Number of iterations, for each original and simulated dataset (1st percentile, 25th percentile, 75th percentile, 99th percentile, mean, and median), to reach the inversion model convergence.

Dataset	T1-DD-200V	T1-DD-400V	T1-DD-400V	T1-DD-Vp800mV	T1-DD-Vpsaven	T1-PD-800V	T1-PD-Vpsaven
original	5	6	6	6	6	4	4
1st perc	12	12	12	12	12	9	10
25th perc	7	7	7	7	7	6	9
75th perc	8	8	8	8	8	6	9
99th perc	14	14	14	14	14	10	11
mean	6	6	6	6	6	5	8
median	5	5	5	5	5	4	7
